# Change in employment status and its causal effect on suicidal ideation and depressive symptoms: A marginal structural model with machine learning algorithms

**DOI:** 10.5271/sjweh.4150

**Published:** 2024-04-01

**Authors:** Jaehong Yoon, Ji-Hwan Kim, Yeonseung Chung, Jinsu Park, Ja-Ho Leigh, Seung-Sup Kim

**Affiliations:** 1 Department of Rehabilitation Medicine, Seoul National University Hospital, Seoul, Republic of Korea.; 2 National Traffic Injury Rehabilitation Research Institute, National Traffic Injury Rehabilitation Hospital, Yangpyeong, Republic of Korea.; 3 Institute of Health and Environment, Seoul National University, Seoul, Republic of Korea.; 4 Department of Mathematical Sciences, Korea Advanced Institute of Science and Technology, Daejeon, South Korea.; 5 Department of Information Statistics, Chungbuk National University, Cheongju, Korea.; 6 Department of Environmental Health Sciences, Seoul National University, Seoul, Republic of Korea.; * These authors contributed equally to this work.

**Keywords:** inverse probability weights, precarious work, social epidemiology, suicide

## Abstract

**Objective:**

This study aimed to assess the causal effect of a change in employment status on suicidal ideation and depressive symptoms by applying marginal structural models (MSM) with machine-learning (ML) algorithms.

**Methods:**

We analyzed data from the 8–15^th^ waves (2013–2020) of the Korean Welfare Panel Study, a nationally representative longitudinal dataset. Our analysis included 13 294 observations from 3621 participants who had standard employment at baseline (2013–2019). Based on employment status at follow-up year (2014–2020), respondents were classified into two groups: (i) maintained standard employment (reference group), (ii) changed to non-standard employment. Suicidal ideation during the past year and depressive symptoms during the past week were assessed through self-report questionnaire. To apply the ML algorithms to the MSM, we conducted eight ML algorithms to build the propensity score indicating a change in employment status. Then, we applied the MSM to examine the causal effect by using inverse probability weights calculated based on the propensity score from ML algorithms.

**Results:**

The random forest algorithm performed best among all algorithms, showing the highest area under the curve 0.702, 95% confidence interval (CI) 0.686–0.718. In the MSM with the random forest algorithm, workers who changed from standard to non-standard employment were 2.07 times more likely to report suicidal ideation compared to those who maintained standard employment (95% CI 1.16–3.70). A similar trend was observed in the analysis of depressive symptoms.

**Conclusions:**

This study found that a change in employment status could lead to a higher risk of suicidal ideation and depressive symptoms.

Standard employment is defined as having a full-time job with the expectation of continued employment at the employer’s place of business (1). According to the Organization for Economic Cooperation and Development (OECD), non-standard employment, including temporary contracts or part-time work, accounted for more than one-third of total employment among OECD countries (2). In South Korea (hereafter Korea), for example, the number of non-standard workers increased from 4.6 million in 2003 to 7.4 million in 2020 (3). Additionally, the proportion of temporary employment in Korea was 26.1% of dependent employment in 2021, which is the second-highest among all OECD countries (4).

There has been a growing body of evidence of an association between employment status and mental health (5, 6), but previous studies have yielded inconsistent results (7–12). Some prior studies reported that non-standard employment may be related to poor mental health, such as depressive symptoms (7, 11, 12) and suicidal ideation (10, 12), while other studies have not found such a link. For example, a cohort study of 107 828 employees in Finland found no association between temporary employment and depression-related work disability (9). Also, a longitudinal study in the UK reported that non-standard employment did not appear to be related to poor mental health when they examined the relationship via a fixed-effect logistic regression (8).

A few studies closely examined the causal effect of non-standard employment on health using propensity score methods (13–15). For example, a longitudinal study of 3577 workers in the US that used propensity score matching found that temporary workers were more likely to report higher depressive symptom scores compared to standard workers (13). Another study of 11 284 waged workers in Spain found a statistically significant increase in poor mental health induced by temporary employment among male workers using propensity score matching (15). In these studies, a standard logistic regression was used to estimate the propensity scores. However, one potential limitation of logistic regression is that it assumes linearity in the association between occupational exposure and related covariates. Such an assumption may result in a biased estimation of the propensity score in that the balance in covariate distribution is not guaranteed if the relationship is more complicated than linear.

It has been suggested that machine-learning (ML) algorithms can be used in causal inference to better estimate propensity scores (16, 17) as they relax the restrictive assumption of linearity and model more complex association between occupational exposure and covariates. One simulation study demonstrated that logistic regression showed poor performance in estimating the propensity score leading to biased inference for causal effect when the true relation between exposure and covariates are moderately non-linear and non-additive (16). With this regard, they suggested that ML algorithms can serve as an alternative to traditional logistic regression for estimating the propensity score, ensuring covariate balance between exposure groups (16, 18, 19). In other words, inverse probability weights estimated with ML algorithms can reduce the risk that the exchangeability assumptions fail due to model misspecification in the estimation of the propensity scores.

However, most studies that examined the causal relationship between non-standard employment and health have adopted logistic regression for estimating the propensity score (13–15, 20). Therefore, this study aimed to assess the causal effect of a change in employment status on suicidal ideation and depressive symptoms by applying a marginal structural model (MSM) with ML algorithms using a longitudinal dataset.

## Methods

### Study population

This study analyzed data from the Korea Welfare Panel Study (KOWEPS), a nationally representative longitudinal dataset. The KOWEPS was launched by the Korean Institute of Social and Health Affairs in 2006. Survey data were collected through person-to-person interviews and the data included 18 856 participants from 7072 households in Korea in the first wave. To date, all of the KOWEPS data from waves 1–15 (2006–2020) are publicly available online (www.kowpes.re.kr). Our analysis included data from the 8–15^th^ waves (2013–2020) of KOWEPS.

**Figure 1:**
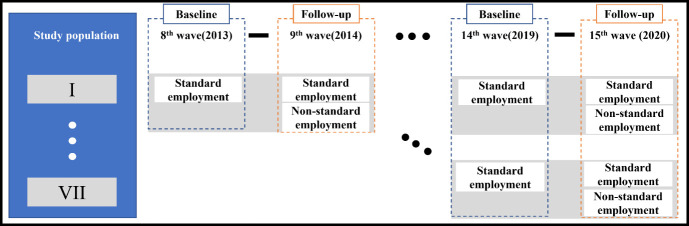
Flow-chart of the study population.

The study population comprised wage workers ≥19 years old with standard employment at baseline (2013–2019). Also, only those who had standard or non-standard employment at follow-up (2014–2020) were included in the analysis. After excluding those who had any missing information with regard to the experience of suicidal ideation, depressive symptoms, employment status, and covariates at baseline, a total of 13 294 observations from 3621 participants were used in the analysis (figure 1). Because KOWEPS is a publicly available dataset, the study received Institutional Review Board (IRB) exemption from the Office of Human Research Administration at Korea University (IRB-2021-0329).

### Measures

*Change in employment status.* Employment status was classified into two categories: standard and non-standard employment. Standard employment was defined as the presence of a contract that met all four of the following criteria: (i) ≥1 year contract duration, (ii) full-time position (not part-time workers), (iii) directly hired by their employer (not subcontracted or dispatched workers or self-employed workers without employees), and (iv) no fixed term in their employment contract. Workers who did not meet any of these four conditions were defined as those in non-standard employment.

Changes in employment status among those in standard employment at baseline (2013–2019) were categorized into two groups based on their employment status in the following year (2014–2020): standard employment (reference group), and non-standard employment. We created seven populations, each consisting of a baseline year (2013, 2014, 2015, 2016, 2017, 2018, and 2019) and its corresponding follow-up year (2014, 2015, 2016, 2017, 2018, 2019, and 2020 respectively). As a result, participants could be repeatedly included up to seven times in the analysis. For example, individuals who remained in standard employment from 2013 to 2020 year would be included seven times in the analysis.

*Suicidal ideation.* Suicidal ideation has been measured annually from waves 8–15 (2013–2020) by asking, “Have you ever seriously thought about dying by suicide over the past year?” (yes or no response). As an outcome variable, suicidal ideation at each follow-up year (2014–2020) was used. Suicidal ideation at baseline (2013–2019) was included in prediction model as a covariate.

*Depressive symptoms.* Depressive symptoms over the past week were assessed using the 11-item version of the Centers for Epidemiologic Studies Depression Scale. Participants rated the frequency of symptoms on a four-point scale, ranging from ‘rarely (<1 day/week)’ to ‘most (≥5 days/week)’ for each of the 11 items. The summed score ranged was 0–33, with higher scores indicating a higher level of depressive symptoms. As an outcome, depressive symptoms at each follow-up year (2014–2020) were used. Baseline depressive symptoms (2013–2019) were included in prediction model as a covariate.

### Covariates

We selected variables to be included in the prediction model for changes in employment status. These were sociodemographic variables (age, sex, region, the number of household members, marital status, educational attainment, occupation, household income, satisfaction level, type of house occupancy, and year at baseline), work-related variables (enterprise size, working hours per week, job satisfaction level, labor union membership, and worker’s compensation insurance), health-related variables (disabilities, chronic diseases, self-rated health, depressive symptoms, and suicidal ideation), and life-related variables (leisure satisfaction level, life satisfaction level, and presence of personal pension) at baseline (2011–2019 year). Age and the number of household members were measured as continuous variables. Sex is defined as male or female. Region was classified into urban areas and other. Marital status was classified as currently, never or previously married, including widowed and divorced. Educational attainment was categorized into four groups (ie, junior high or less, high school graduate, college graduate, university graduate or more). Equivalized household income was calculated by dividing household income by the square root of the number of household members and log-transformed for the analysis. The household income satisfaction level was measured by the question, “How satisfied are you with your household income?” Responses ranged from “very satisfied” (score 1) to “very dissatisfied” (score 5) and were dichotomized into satisfaction (for responses 1–3) and dissatisfaction (for responses 4–5). House occupancy was divided into four groups (ie, house owner, *jeonse –* a type of housing/building lease in Korea, where the lessee pays the a lump sum deposit (21), monthly rent, and other). Occupation was categorized into eight groups (ie, senior manager, professional/technical, clerical, service, sales, skilled, machine operator, and unskilled).

For work-related variables, enterprise size was divided into four categories (1–4, 5–49, 50–99, 100–299 workers, and ≥300 workers). Working hours per week were measured as a continuous variable. Job satisfaction level was measured by the question, “How satisfied are you with your job?” Responses ranged from “very satisfied” (score 1) to “very dissatisfied” (score 5) and were dichotomized into satisfaction (for responses 1–3) and dissatisfaction (for responses 4–5). Labor union membership was coded into four groups: union members, not union members at a workplace with a labor union, workers who were not eligible to take membership at their workplace with a labor union, and workers at workplaces without a labor union. Worker’s compensation insurance was categorized into three groups (ie, workers with worker’s compensation insurance, workers without worker’s compensation insurance, and workers who were not eligible to get worker’s compensation insurance).

For health-related variables, disabilities, chronic diseases, and suicidal ideation were divided into two groups: yes or no. Self-rated health was assessed on a five-point scale with the question “How would you rate your overall health?” Responses ranged from “very good” (score 1) to “very poor” (score 5) and were dichotomized into good health (for responses 1–3) and poor health (for responses 4–5). Depressive symptoms at baseline were included as a continuous variable. For life-related variables, each satisfaction level for leisure and life was divided into satisfaction and dissatisfaction. The presence of personal pension was classified into two groups (yes or no).

### Analysis

The ML algorithms were applied to the MSM in two steps (supplementary material, www.sjweh.fi/article/4150, figure S1). First, we evaluated eight different ML algorithms to build the model to predict the propensity score of a change in employment status. These were logistic regression, random forest, penalized logistic regression (lasso, ridge, and elastic net), support vector machine (radial basis function and polynomial function) and single-layer artificial neural networks. Supplementary table S1 details the tuning parameters for each ML algorithm. We applied ten-fold cross-validation to identify the optimal tuning parameters for each algorithm and assessed their predictive performance capabilities using the area under the curve (AUC) of the receiver operating characteristic (ROC) curve. We selected the algorithm with the largest AUC as that which performed best.

Second, we applied the MSM to examine the causal effect of a change in employment status on suicidal ideation and depressive symptoms using inverse probability weight (IPW). The IPW makes it possible to estimate a causal association by creating a pseudo-population in which exposure is not associated with covariates. Furthermore, we used stabilized IPW to consider the inflated sample size in the pseudo-population.

Finally, we utilized a random intercept logistic and linear model in the pseudo-population to control autocorrelation between observations within the same individuals. In the analysis, age, the number of household members, household income, working hours per week, and depressive symptoms were included as continuous variables. All other covariates were included as categorical variables. Results from the MSM with stabilized IPW using ML algorithms are presented as odds ratios (OR) and coefficient (β) with 95% confidence intervals (CI) using STATA/MP (Stata Corp, College Station, TX, USA, version 17.0). All ML algorithms were estimated from the *tidymodels* package in R statistical software (version 4.0.2; R Development Core Team).

It should be noted that our estimates from MSM would be interpreted as causal given the following assumptions (22, 23): consistency (ie, observed outcome for every treated/untreated individual equals their outcome if they had been treated/untreated), exchangeability (ie, the risk of outcome in treated individuals is the same as the risk of outcome in untreated individuals when they would be treated), and positivity (ie, both treated and untreated individuals exist at every level of confounders). To ascertain approximate exchangeability of our MSM with ML algorithms, we conducted two post-hoc analysis. First, we estimated the standardized mean differences in each covariate used in prediction model between workers who maintained standard employment (non-exposed group) and those who experienced a change in employment status (exposed group) for checking covariate balance. Previous studies have reported that a standardized mean difference >0.1 indicates a remaining imbalance in covariates between groups (24, 25). Second, to reduce residual confounding, we used a double-robust model that incorporated both the ML-based IPW and an adjustment for covariates included as predictors for ML algorithms.

## Results

Table 1 presents the distribution of the study population and the change in employment status by covariates among the standard workers at baseline. Overall, 10.8% of standard workers at baseline were in non-standard employment in the follow-up year. Changes in employment status were more common among female, those who were previously married, had lower educational level, worked in service workers, and were dissatisfied with household income. Additionally, people working in small-sized enterprises, dissatisfied with their jobs, lacking labor union representation at their workplace, without worker’s compensation, having chronic diseases, having suicidal ideation, reporting poor self-rated health, dissatisfied with leisure and life, and without personal pension membership showed a higher prevalence of change in their employment status.

**Table 1:** Distribution of study population and change in employment status by covariates among standard workers at baseline in South Korea (N=13 294). [SD=standard deviation.]

Variable		Total population			Change from standard to non-standard employment		P-value
		N (%)	Mean (SD)		N (%)	Mean (SD)	
**Total**		13 294 (100.0)			1441 (10.8)		
**Socio-demographic variables**							
Sex							<0.001 ^a^
	Male	8507 (64.0)			782 (9.2)		
	Female	4787 (36.0)			659 (13.8)		
Age			41.3 (9.9)			42.6 (11.9)	<0.001 ^b^
Number of house- hold members			3.4 (1.2)			3.3 (1.2)	<0.001 ^b^
Region							0.738 ^a^
	Urban	12 053 (90.7)			1303 (10.8)		
	Rural	1 241 (9.3)			138 (11.1)		
Marital status							<0.001 ^a^
	Currently married	9648 (72.6)			938 (9.7)		
	Previously married	676 (5.1)			124 (18.3)		
	Never married	2970 (22.3)			379 (12.8)		
Educational attainment							<0.001 ^a^
	Junior high or less	792 (6.0)			165 (20.8)		
	High school graduate	4619 (34.7)			624 (13.5)		
	College graduate	2364 (17.8)			244 (10.3)		
	University graduate or higher	5519 (41.5)			408 (7.4)		
Occupation							<0.001 ^a^
	Senior manager	625 (4.7)			60 (9.6)		
	Professional/technical	3234 (24.3)			305 (9.4)		
	Clerical	3920 (29.5)			302 (7.7)		
	Service	774 (5.8)			147 (19.0)		
	Sales	777 (5.8)			102 (13.1)		
	Skilled	1196 (9.0)			122 (10.2)		
	Machine operator	1578 (11.9)			181 (11.5)		
	Unskilled	1190 (9.0)			222 (18.7)		
Household income			8.2 (0.4)			8.1 (0.5)	<0.001 ^b^
Satisfaction level of household income							<0.001 ^a^
	Satisfied	10 274 (77.3)			992 (9.7)		
	Dissatisfied	3020 (22.7)			449 (14.9)		
House occupancy							<0.001 ^a^
	House owner	8218 (61.8)			819 (10.0)		
	Jeonse	2585 (19.4)			275 (10.6)		
	Monthly renter	1862 (14.0)			268 (14.4)		
	Other	629 (4.7)			79 (12.6)		
Year at baseline							0.016 ^a^
	2013	1998 (15.0)			249 (12.5)		
	2014	1915 (14.4)			211 (11.0)		
	2015	1932 (14.5)			209 (10.8)		
	2016	1899 (14.3)			193 (10.2)		
	2017	1923 (14.5)			178 (9.3)		
	2018	1833 (13.8)			184 (10.0)		
	2019	1794 (13.5)			217 (12.1)		
**Work-related variables**							
Enterprise size (workers)							<0.001 ^a^
	1–49	5814 (43.7)			841 (14.5)		
	50–69	1328 (10.0)			165 (12.4)		
	100–299	1603 (12.1)			152 (9.5)		
	≥300	4549 (34.2)			283 (6.2)		
Working hours per week			45.0 (9.3)			45.9 (10.5)	<0.001 ^b^
Satisfaction level of job							<0.001 ^a^
	Satisfied	12 457 (93.7)			1303 (10.5)		
	Dissatisfied	837 (6.3)			138 (16.5)		
Union labor membership							<0.001 ^a^
	Yes	2425 (18.2)			149 (6.1)		
	No (at workplace with labor union)	1251 (9.4)			82 (6.6)		
	Not eligible (at workplace with labor union)	833 (6.3)			60 (7.2)		
	No union at workplace	8785 (66.1)			1150 (13.1)		
Workers' comp							<0.001 ^a^
	Yes	10 840 (81.5)			1200 (11.1)		
	No	557 (4.2)			167 (30.0)		
	Not eligible	1897 (14.3)			74 (3.9)		
**Health-related variables**							
Disability							0.250 ^a^
	No	12 923 (97.2)			1394 (10.8)		
	Yes	371 (2.8)			47 (12.7)		
Chronic diseases							<0.001 ^a^
	No	9543 (71.8)			952 (10.0)		
	Yes	3751 (28.2)			489 (13.0)		
Suicidal ideation at baseline							<0.001 ^a^
	No	13 160 (99.0)			1409 (10.7)		
	Yes	134 (1.0)			32 (23.9)		
Depressive symptoms at baseline			1.7 (3.0)			2.2 (3.5)	<0.001 ^b^
Self-rated health							<0.001 ^a^
	Good	12 925 (97.2)			1377 (10.7)		
	Poor	369 (2.8)			64 (17.3)		
**Life-related variables**							
Satisfaction level of leisure							<0.001 ^a^
	Satisfied	11 489 (86.4)			1179 (10.3)		
	Dissatisfied	1805 (13.6)			262 (14.5)		
Satisfaction level of life							<0.001 ^a^
	Satisfied	12 975 (97.6)			1383 (10.7)		
	Dissatisfied	319 (2.4)			58 (18.2)		
Personal pension membership							<0.001 ^a^
	No	6995 (52.6)			940 (13.4)		
	Yes	6299 (47.4)			501 (8.0)		

^a^ P-value of the chi-square test comparing the prevalence of change from standard employment to non-standard employment across the different groups. ^b^ P-value of the t-test comparing the mean of covariates across change from standard employment to non-standard employment status.

**Figure 2:**
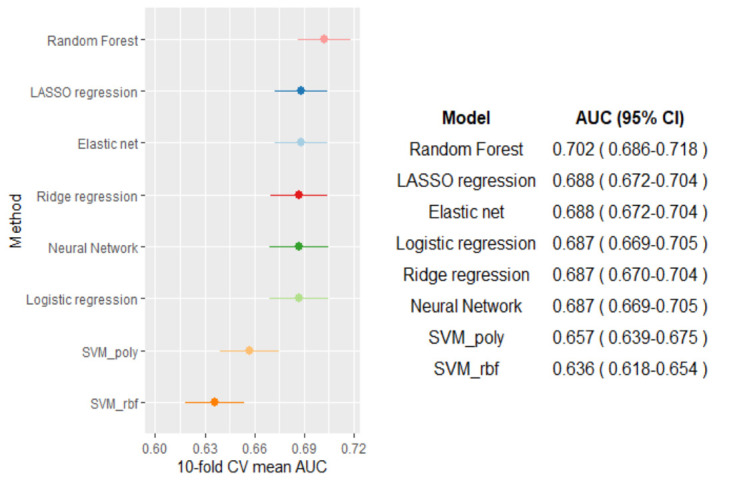
Cross-validated performance of the machine learning algorithms according to the AUC.

Figure 2 shows the cross-validated AUC for each of the eight ML algorithms. While the AUC for each algorithm ranged from 0.636–0.702, the random forest algorithm showed the largest AUC (AUC 0.702, 95% CI 0.686–0.718). Table 2 shows the relationship between a change in employment status and suicidal ideation. In the marginal structural model which used the random forest algorithm to estimate IPW, a change from standard to non-standard employment was associated with a higher risk of suicidal ideation (OR 2.07, 95% CI 1.16– 3.70) and depressive symptoms (β 0.41, 95% CI 0.23–0.59) (table 3).

**Table 2:** Association between change of employment and suicidal ideation in Korea (N=13 294). [OR=odds ratio; CI=confidence interval.]

Change in employment status		Distribution		Prevalence of suicidal ideation		Model 1^a^			Model 2^b^	
		N (%)		N (%)		OR	95% CI		OR	95% CI
Baseline	Follow-up									
Standard	Standard	11 853 (89.2)		87 (0.7)		1.00	Referent		1.00	Referent
	Non-standard	1441 (10.8)		25 (1.7)		2.07 *	(1.16–3.70)		1.97 *	(1.11–3.48)

^a^ Model 1: Marginal structural model with stabilized IPW estimated using random forest. ^b^ Model 2: Model 1+ adjusted for all covariates used in random forest prediction model for estimating stabilized IPW. *P<0.05.

**Table 3:** Association between change of employment and depressive symptoms in Korea (N=13 294). [CI=confidence interval.]

Change in employment status		Distribution		Depressive symptoms		Model 1 ^a^			Model 2 ^b^	
		N (%)		Mean (SD)		Coefficient	95% CI		Coefficient	95% CI
Baseline	Follow-up									
Standard	Standard	11 853 (89.2)		1.7 (2.9)		Referent	Referent		Referent	Referent
	Non-standard	1441 (10.8)		2.3 (3.2)		0.41***	(0.23-0.59)		0.37***	(0.19-0.55)

^a^ Model 1: Marginal structural model with stabilized IPW estimated using random forest. ^b^ Model 2: Model 1+ adjusted for all covariates used in random forest prediction model for estimating stabilized IPW. ***P<0.001.

**Figure 3:**
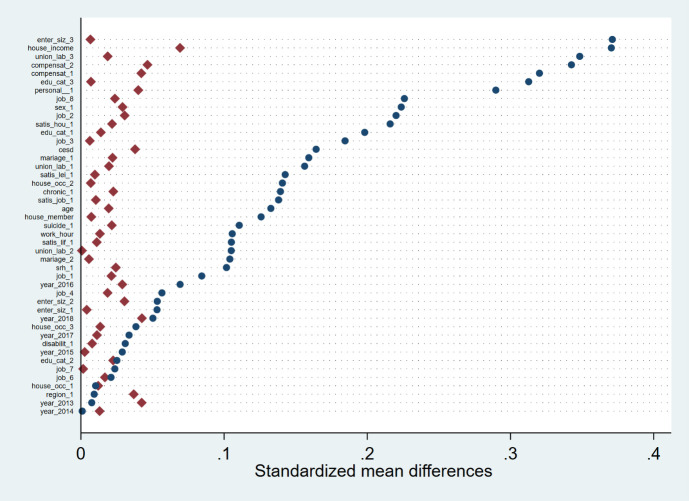
Balance in the unweighted sample (blue circle) and weighted sample with weights generated by the random forest (red diamond).

The MSM with stabilized IPW estimated using random forest improves the balance of most covariates compared to the raw population (figure 3). In the pseudo-population, a standardized mean difference was <0.1 for all covariates. It was observed that the standardized mean difference was higher in the pseudo-population than in the raw population for some covariates, including house owner, and urban area. After adjusting for covariates at baseline in the pseudo-population, the relationship between a change in employment status and suicidal ideation was attenuated but remained statistically significant (OR 1.97, 95% CI 1.11–3.48) (table 2). Similarly, the association was also attenuated and still statistically significant in double-robust model for depressive symptoms (β 0.37, 95% CI 0.19–0.55) (table 3).

## Discussion

In this study, we built a prediction model using ML algorithms to estimate the propensity score of a change in employment status. We found a causal relationship of change in employment status with suicidal ideation and depressive symptoms using a MSM with ML algorithms. Workers who changed from standard to non-standard employment were more likely to experience suicidal ideation and depressive symptoms than those who maintained standard employment. These results are consistent with those from previous studies reporting that a change in employment status may be related to poor mental health (10, 26, 27). For example, a longitudinal study showed that people who became non-standard workers had higher odds of depressive symptoms than those who maintained standard employment after adjusting for baseline depressive symptoms (27). Another longitudinal study reported that workers who changed from standard to non-standard employment were more likely to experience suicidal ideation than those who maintained standard employment after excluding those who had baseline suicidal ideation (10).

The higher risk of suicidal ideation and depressive symptoms among people who changed into non-standard employment could be explained by job insecurity and poor working conditions (28–30). A systematic review study argued precarious employment characterized by low job security has an adverse effect on workers’ mental health (5). Also, a previous study of Korean workers found that those with non-standard employment were more likely to be exposed to physical, chemical, and ergonomic hazards at workplace, compared to those with standard employment (31). Furthermore, a qualitative study reported that the feeling of mistrust for being protected by labor unions when they needed help may be a potential mechanism linking temporary employment and poor mental conditions because most temporary workers do not belong to labor unions (32). According to a Korea Labor & Society Institute report in 2020, the labor union membership rate among non-standard workers was 2.6%, while that among standard workers was 19.2% (33). Furthermore, a meta-analysis of 27 studies showed that temporary employment was related to a higher risk of occupational injuries and lower sickness absence rates (29). This suggests that temporary workers may be unable to take sick leave, which could have a negative influence on their mental health.

This study applied MSM with ML algorithms to assess the causal relationship of change in employment with suicidal ideation and depressive symptoms. We fitted eight different ML algorithms to predict the propensity score and the random forest method showed the best prediction performance with the largest AUC value. Nevertheless, other methods including the standard logistic regression performed almost comparably showing slightly smaller AUC values (34). This implies that the actual relationship between the change in employment status and related covariates may be almost linear and not much interactive, so using the MSM with a standard logistic regression, which is the traditional approach in IPW method, would lead to similar results and conclusions in the current dataset. However, in general, the true relationship between exposure and related covariates is unknown a priori in epidemiological studies. Considering more general methods like ML algorithms would be beneficial as they can reveal non-linear and non-additive relationship while including simpler models assuming linearity and additivity as special cases. Furthermore, the ML methods can be implemented easily using freely available software nowadays. Therefore, it is worth to use ML algorithms in future occupational epidemiology studies as they offer more advantages than logistic regression in estimating IPW, particularly when the relationship between occupational exposures and other covariates is non-linear and complex.

Some limitations should be noted in this study. First, we could not be free from the possibility of reverse causality, especially in the analysis of suicidal ideation, because change in employment status and suicidal ideation over the past year were measured in the same wave of survey. It is possible that workers had a suicidal ideation before they became non-standard workers. However, the results from analysis of depressive symptoms are less likely to be vulnerable to reverse causality because the symptoms were assessed during the past week.

Second, even though we used 24 variables and checked eight ML algorithms to predict inverse probability weights and applied double-robust model in the data analysis, there might still be unmeasured residual confounding affecting the causal impact of change in employment status on suicidal ideation and depressive symptoms. For example, information pertaining to the employment rate in the administrative district was not measured in the KOWEPS, though it could be associated with worker’s tendency of changing into non-standard employment. So we cannot completely rule out the possibility of violating exchangeability assumptions for our estimates from MSM interpreted as casual.

Third, there was a possibility of follow-up loss. For example, people who experienced suicidal ideation at baseline may not have participated in the survey in the follow-up year. We compared the distribution of covariates between our study population and the population with standard employment at baseline (supplementary table S2). The distribution was similar between the two groups, but the subjects included in the study population were more likely to be married and have satisfied life and leisure. Fourth, we did not consider reasons for changes in employment status. Cuyper & Witte (35)reported that people who voluntarily changed their employment status from standard to non-standard were more likely to report a higher level of life satisfaction. Therefore, future studies should consider the reasons of change in employment status.

### Concluding remarks

In conclusion, our study has revealed that change from standard to non-standard employment may increase the risk of suicidal ideation and depressive symptoms among Korean workers. Notably, this is the first study to assess the causal effects of employment status changes on suicidal ideation and depressive symptoms using a MSM combined with ML algorithms. Furthermore, our research introduces a strategy for applying ML algorithms to occupational epidemiology studies aimed at investigating the causal effects of occupational exposures on workers’ health.

## Supplementary material

Supplementary material
