# Supplementary material for: Change in employment status and its causal effect on suicidal ideation and depressive symptoms: A marginal structural model with machine learning algorithms
Source: Scand J Work Environ Health. 2024 Mar 27;50(3):218–27. doi: 10.5271/sjweh.4150 (PMC11106614; doi:10.5271/sjweh.4150)
Supplement: Supplementary material [file SJWEH-50-218-S001.pdf]

Change in employment status and its causal effect on suicidal ideation and depressive symptoms: A marginal structural model with machine learning algorithms[1]

by Jaehong Yoon, PhD, Ji-Hwan Kim, PhD, Yeonseung Chung, PhD, Jinsu Park, PhD, Ja-Ho Leigh, MD, MSc, Seung-Sup Kim, MD, MPH, ScD [2]

1. Supplementary material
2. Correspondence to: Seung-Sup Kim, Department of Environmental Health Sciences, Seoul National University, Room 718, Bldg 220, Gwanak-ro 1, Seoul 08826, Republic of Korea. [E-mail: kim.seungsup@snu.ac.kr]

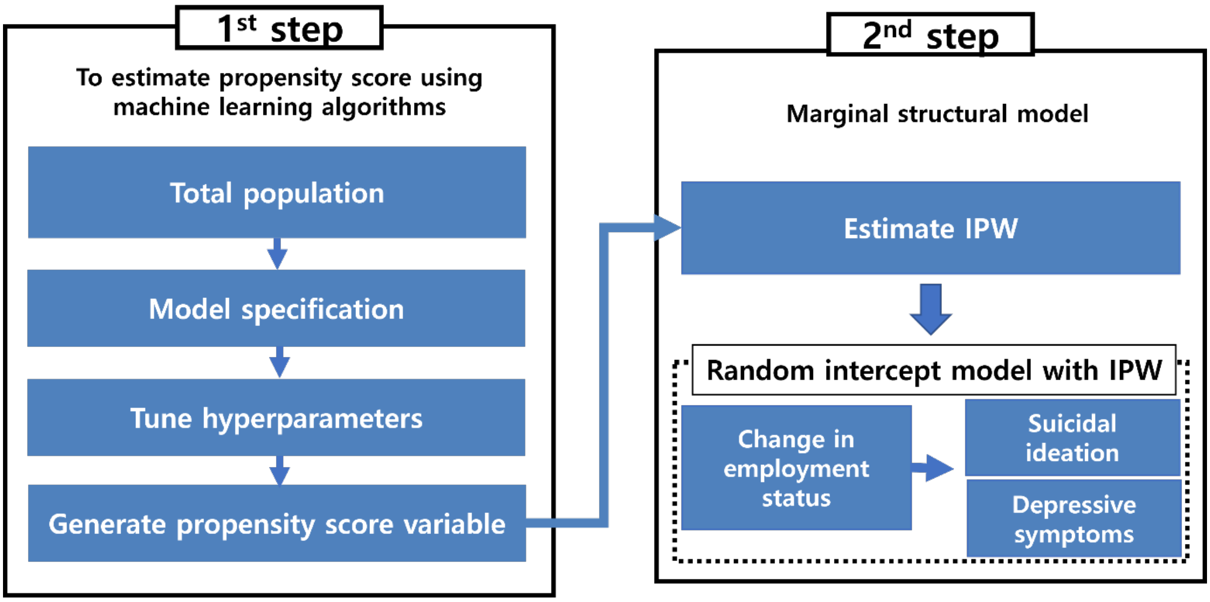

Figure S1. Flow chart of analysis

Table S1. Machine learning algorithms and tuning parameters for each model

Algorithms	Tuning parameters
Logistic regression	
Random Forest	mtry (the number of predictors that will be randomly sampled at each split when creating the tree models), min_n (the minimum number of data points in a node that is required for the node to be split further)
Ridge regression	penalty (the total amount of regularization)
LASSO regression	penalty (the total amount of regularization)
Elastic net	penalty (the total amount of regularization), mixture (the proportion of lasso regularization in the model)
Support Vector Machine with radial basis function	cost (the cost of predicting a sample within or on the wrong side of the margin), rbf_sigma (A positive number for radial basis function), margin (epsilon in the SVM insensitive loss function)
Support Vector Machine with polynomial function	cost (cost of predicting a sample within or on the wrong side of the margin), scale_factor (polynomial scaling factor), margin (epsilon in the SVM insensitive loss function), degree (polynomial degree)
Neural networks	hidden_units (the number of units in the hidden model.), dropout (the proportion of model parameters randomly set to zero during model training), penalty (the amount of weight decay)

Table S2. The distribution of total population with standard employment at baseline and total population included in this research by covariates

Variable	Total population with standard employment at baseline		Total population included in the study	
	N (%)	Mean (SD)	N (%)	Mean (SD)
**Total**	16 324 (100.0)		13 294 (100.0)	
**Sociodemographic variables**				
**Sex**				
Male	10 416 (63.8)		8507 (64.0)	
Female	5908 (36.2)		4787 (36.0)	
**Age**		41.2 (10.4)		41.3 (9.9)
**The number of household members**		3.4 (1.2)		3.4 (1.2)
**Region**				
Urban	14 757 (90.4)		12 053 (90.7)	
Rural	1567 (9.6)		1241 (9.3)	
**Marital status**				
Currently married	11 499 (70.4)		9648 (72.6)	
Previously married	805 (4.9)		676 (5.1)	
Never married	4020 (24.6)		2970 (22.3)	
**Educational attainment**				
Junior high or less	907 (5.6)		792 (6.0)	
High school graduate	5603 (34.3)		4619 (34.7)	
College graduate	2989 (18.3)		2364 (17.8)	
University graduate or more	6825 (41.8)		5519 (41.5)	
**Occupation**				
Senior manager	785 (4.8)		625 (4.7)	
Professional/technical	3959 (24.3)		3234 (24.3)	
Clerical	4747 (29.1)		3920 (29.5)	
Service	990 (6.1)		774 (5.8)	
Sales	928 (5.7)		777 (5.8)	
Agriculture/fisheries	34 (0.2)		0 (0.0)	
Skilled	1481 (9.1)		1196 (9.0)	
Machine operator	1851 (11.3)		1578 (11.9)	
Unskilled	1408 (8.6)		1190 (9.0)	
Missing	141 (0.9)		0 (0.0)	

**Household income**		8.3 (0.4)		8.2 (0.4)
**Satisfcation level of household income**				
Satisfaction	12 505 (76.6)		10 274 (77.3)	
Dissatisfaction	3137 (19.2)		3020 (22.7)	
Missing	682 (4.2)		0 (0.0)	
**House_occupancy**				
House owner	10 156 (62.2)		8218 (61.8)	
Jeonse	3038 (18.6)		2585 (19.4)	
Monthly rent	2301 (14.1)		1862 (14.0)	
Others	735 (4.5)		629 (4.7)	
Missing	94 (0.6)		0 (0.0)	
**Year at baseline**				
2013	2370 (14.5)		1998 (15.0)	
2014	2430 (14.9)		1915 (14.4)	
2015	2375 (14.5)		1932 (14.5)	
2016	2357 (14.4)		1899 (14.3)	
2017	2298 (14.1)		1923 (14.5)	
2018	2305 (14.1)		1833 (13.8)	
2019	2189 (13.4)		1794 (13.5)	
**Work-related variables**				
**Enterprise size**				
1-49 workers	7239 (44.3)		5814 (43.7)	
50-69 workers	1652 (10.1)		1328 (10.0)	
100-299 workers	1983 (12.1)		1603 (12.1)	
300 workers or more	5441 (33.3)		4549 (34.2)	
Missing	9 (0.1)		0 (0.0)	
**Working hours per week**		44.5 (8.9)		45.0 (9.3)
**Satisfaction level of job**				
Satisfaction	14 706 (90.1)		12 457 (93.7)	
Dissatisfaction	936 (5.7)		837 (6.3)	
Missing	682 (4.2)		0 (0.0)	
**Union labor membership**				
Workers with union membership	2764 (16.9)		2425 (18.2)	
Workers without union membership at workplace with labor union	1511 (9.3)		1251 (9.4)	
Workers who were not eligible to join the membership at workplace with labor union	1023 (6.3)		833 (6.3)	

Workers at workplace without labor union	11026 (67.5)	8785 (66.1)
**Worker's compensation membership**		
Workers with worker's compensation membership	13362 (81.9)	10840 (81.5)
Workers without compensation membership	676 (4.1)	557 (4.2)
Workers who were not eligible to join the worker's compensation membership	2286 (14.0)	1897 (14.3)
**Health-related variables**		
**Disability**		
No	15 863 (97.2)	12 923 (97.2)
Yes	461 (2.8)	371 (2.8)
**Chronic diseases**		
No	11 705 (71.7)	9543 (71.8)
Yes	4619 (28.3)	3751 (28.2)
**Suicidal ideation at baseline**		
No	15 222 (93.2)	13 160 (99.0)
Yes	132 (0.8)	134 (1.0)
Missing	970 (5.9)	0 (0.0)
**Depressive symptoms at baseline**	1.7 (2.9)	1.7 (3.0)
**Self-rated health**		
Good	15863 (97.2)	12925 (97.2)
Poor	461 (2.8)	369 (2.8)
**Life-related variables**		
**Satisfaction level of leisure**		
Satisfaction	13 746 (84.2)	11 489 (86.4)
Dissatisfaction	1896 (11.6)	1805 (13.6)
Missing	682 (4.2)	0 (0.0)
**Satisfaction level of life**		
Satisfaction	15 307 (93.8)	12 975 (97.6)
Dissatisfaction	335 (2.1)	319 (2.4)
Missing	682 (4.2)	0 (0.0)
**Personal pension membership**		
No	9077 (55.6)	6995 (52.6)
Yes	7247 (44.4)	6299 (47.4)
